# Harnessing SARS-CoV-2 immunity to promote antitumor responses through intratumoral vaccination and adoptive transfer

**DOI:** 10.3389/fimmu.2026.1711569

**Published:** 2026-02-23

**Authors:** Manar Darwish, Mais Eyouni, Mohammed Yassir Khan, Rofidah Alsaggaf, Rahaf Alharbi, Haya Hawbani, Rwaa H. Abdulal, Rowa Alhabbab, Ala A. Azhari, Tarfa Altorki, May Alsayb, Heba M. Zahid, Mustafa Taher, Almohanad Alkayyal, Anwar M. Hashem, Ahmad Bakur Mahmoud

**Affiliations:** 1 Vaccines and Immunotherapy Unit, King Fahd Medical Research Center, King Abdulaziz University, Jeddah, Saudi Arabia; 2 Infectious Disease Research Department, King Abdullah International Medical Research Center, King Saud bin Abdulaziz University for Health Sciences, Ministry of National Guard Health Affairs, Jeddah, United States; 3 Department of Medical Laboratory Technology, Faculty of Applied Medical Sciences, King Abdulaziz University, Jeddah, Saudi Arabia; 4 Department of Clinical Microbiology and Immunology, Faculty of Medicine, King Abdulaziz University, Jeddah, Saudi Arabia; 5 Health and Life Research Center, Taibah University, Madinah, Saudi Arabia; 6 Department of Clinical Laboratory Sciences, College of Applied Medical Sciences, Taibah University, Madinah, Saudi Arabia; 7 Department of Medical Laboratory Technology, Faculty of Applied Medical Sciences, University of Tabuk, Tabuk, Saudi Arabia

**Keywords:** adoptive transfer, cancer immunotherapy, cancer vaccine, DNA vaccine, SARS-CoV-2 immunity

## Abstract

**Introduction:**

Cancer immunotherapy holds promise for the treatment of malignancies by mobilizing the immune system; however, its efficacy remains limited in tumors that lack immune infiltration. Innovative approaches are therefore required to convert these immunologically “cold” tumors into “hot” tumors that are more susceptible to immune-mediated attack. Therapeutic vaccination represents one such strategy, capable of triggering robust immune activation and durable memory responses. Given that the vast majority of the global population has either been vaccinated against or exposed to SARS-CoV-2, we hypothesized that pre-existing antiviral immunity could be locally leveraged to enhance intratumoral immune responses.

**Methods:**

To test this concept, we evaluated the therapeutic efficacy of intratumoral SARS-CoV-2 vaccination in the B16F10 murine melanoma model using two SARS-CoV-2 vaccine platforms: pVAX-SARS-S, a DNA vaccine encoding the spike protein S1 subunit and rAd-SARS2-S1/CD40L, a recombinant adenovirus expressing a secreted S1-CD40L fusion protein. Tumor-bearing C57BL/6 mice were treated intratumorally with either vaccine in the presence or absence of prior immunization, which had been established six months earlier to mimic pre-existing antiviral immunity.

**Results:**

Intratumoral vaccination with pVAX-SARS-S significantly reduced tumor burden and prolonged survival in both naïve and pre-immunized mice. Its antitumor protection could be adoptively transferred via splenocytes, indicating the involvement of systemic adaptive immunity in mediating tumor rejection. The rAd-SARS2-S1/CD40L platform conferred modest tumor control. While both vaccines were effective, the DNA platform demonstrated superior efficacy.

**Discussion:**

Together, these findings provide proof-of-concept that intratumoral administration of SARS-CoV-2 vaccines can promote antitumor effects through local immune activation in the context of pre-existing antiviral immunity. This strategy may offer a translatable approach for enhancing intratumoral immunotherapy in the post-pandemic era.

## Introduction

The field of cancer treatment has undergone significant changes in recent years due to the emergence of immunotherapy as a revolutionary approach. Cancer immunotherapy is a rapidly growing field that focuses on empowering the immune system to target and destroy tumor cells. However, the effectiveness of immunotherapy is limited by the tumor microenvironment (TME), particularly in tumors lacking immune infiltration (1). The TME can act as a barrier to effective immunotherapy by inhibiting the natural immune response. To address this limitation, innovative strategies are required to transform “cold” TMEs into “hot” ones, thereby increasing the infiltration of inflammatory immune cells that serve as targets for immunotherapies (2).

Several strategies have been proposed to reprogram the TME, including the use of immunomodulatory agents that activate the immune system, boost the immune response against the tumor and overcome the immunosuppressive “cold” environment. One such approach involves inducing infection at the tumor site with non-tumor-specific pathogens, thereby generating a robust immune response within the TME that is unrelated to the immune response against cancer, which can serve as a trigger for a strong tumor-specific immune response (3). The site of the tumor, the natural site of viral infection and the methods of providing host viral immunity are all key factors and must be carefully manipulated to elicit an effective immune response within the TME (4).

Vaccination is a promising strategy that can stimulate immune responses and immunological memory, mimicking natural infection. Vaccines provide durable immunity by administering antigens and adjuvants that elicit strong immune responses comparable to those observed following natural infection (5). Repurposing FDA-approved vaccines has been shown to protect against viral infections, increase immune cell infiltration and reduce tumor growth (6, 7). FDA-approved vaccines, such as the SARS-CoV-2 vaccine, can augment antitumor immune responses within the TME. Intratumoral injection of SARS-CoV-2 vaccines can induce localized inflammatory responses at the injection site, reducing local tumor growth, leading to augmented systemic antitumor immunity and improved survival. Earlier studies have revealed improved anti-cancer outcomes by directing pathogens to the tumor site (4, 8–10). In this study, we explored the effect of different SARS-CoV-2 vaccines on tumor growth and survival. Two different SARS-CoV-2 vaccines, pVAX-SARS-S and rAd-SARS-S/CD40L, were used to examine the outcome of the systemic and local SARS-CoV-2 viral immunity on tumor growth and survival in a melanoma mouse model. SARS-CoV-2 was chosen as the model of viral immunity because SARS-CoV-2 infection was recently a major pandemic in the general population and millions of people around the world are currently vaccinated against it. Overall, innovative approaches that enhance immune-mediated responses within the TME hold great promise for improving outcomes in patients with cancer as therapeutic strategies continue to evolve.

## Materials and methods

### Cell culture

The B16F10 murine melanoma cell line (CRL-6475, ATCC) was cultured in Dulbecco’s modified Eagle medium (DMEM) (Thermo Fisher, Waltham, MA) supplemented with penicillin (100 U/mL), streptomycin (100 µg/mL) and 10% heat-inactivated fetal bovine serum (FBS) (Biosera Europe, France). Cells were maintained at 37°C with 5% CO_2_.

Cells were subcultured routinely every three days to ensure cellular vitality and maintain optimal growth conditions. Subculturing was performed one day prior to tumor induction to achieve approximately 80% confluency on the day of induction. Cell count and viability were assessed using trypan blue exclusion and the Countess™ cell counter (Thermo Fisher). Notably, only cell preparations with viability > 90% were used for tumor induction.

### Mice

Female C57BL/6J mice (6–8 weeks old) were obtained from the animal facility at King Fahd Medical Research Center (KFMRC), King Abdulaziz University (KAU), Jeddah, Saudi Arabia.

Experimental procedures adhered to the guidelines approved by the Animal Care and Use Committee (ACUC) at the animal facility, KFMRC. The study protocol was approved by the KFMRC Bioethical Committee at KAU (Approval No. ACUC-20-03-9-9).

### Tumor model and induction

The melanoma model was induced through subcutaneous injection of B16F10 murine melanoma cells. Suspensions containing 3 × 10^5^ B16F10 cells were prepared in 50 µL of sterile phosphate-buffered saline (PBS) per mouse. These injections were administered into the right flank of mice, unless otherwise specified.

### Tumor measurements and survival monitoring

Tumor growth was systematically monitored every three days using digital calipers to measure tumor length and width. Subsequently, tumor volume was calculated using the formula *V = (length × width^2^)/2*. Prior to tumor induction, mice were randomized into distinct treatment groups to ensure unbiased allocation.

Survival was monitored daily for each treatment group. Study endpoints were defined according to approved humane criteria, including tumor-related clinical signs of distress or complications, in accordance with the guidelines approved by the Animal Care and Use Committee (ACUC). Animals were euthanized upon reaching approved humane endpoints (11, 12).

### SARS-CoV-2 vaccines preparation and administration

The pVAX-SARS-S vaccine used in this study was obtained from the Viral and Immunotherapy Unit (VIU) at KFMRC. Bulk endotoxin-free preparations of pVAX-SARS-S (VIU-1005) were performed as described previously (13) using the GenEluteTM HP Select Plasmid Gigaprep Kit (Sigma, Germany). Injections were prepared at a concentration of 1 µg/µL and administered as 100 µg per dose.

The adenoviral vaccine, Ad5-SARS2-S1/tlhCD40L, is a replication-deficient adenovirus vector expressing the SARS-CoV-2 spike protein (rAd-SARS-CoV-2-S) and CD40L. It was purchased from Vector Biolabs, USA, with a concentration of 1.1 × 10¹¹ PFU/mL and prepared into injections of 5 × 10^7^ PFU/mouse in 50 µL PBS.

In the therapeutic group, mice with established melanoma tumors were randomly assigned to treatment and control groups. Intratumoral vaccine injections of either pVAX-SARS-S, rAd-SARS2-S1/CD40L, or PBS were administered three days after tumor implantation and delivered at the tumor implantation site. At early time points, when tumors were not yet palpable, injections were delivered intradermally at the site of tumor cell inoculation; subsequent injections were administered directly into palpable tumors as tumors became established. For the short-term treatment; six doses of each vaccine were administered every three days. For the prolonged treatment; six doses of each vaccine were administered every three days, followed by weekly intratumoral vaccine injections as described in each experiment.

In the prophylactic group, mice were immunized six months before melanoma tumor induction via intramuscular injections of either pVAX-SARS-S, rAd-SARS2-S1/CD40L, or PBS. The pVAX-SARS-S group received three doses at two-week intervals, whereas the rAd-SARS2-S1/CD40L group received two doses with a three-week interval. Subsequently, intratumoral vaccine injections were administered as described in the therapeutic group.

### *In-vivo* rejection assay

Mice were immunized with either pVAX-SARS-S, rAd-SARS2-S1/CD40L, or PBS, followed by tumor induction and intratumoral treatments, as previously described in the short-term treatment. On day 21 post-tumor induction, spleens were harvested and processed.

Single-cell suspensions of splenocytes were prepared from individual immunized and control mice. Briefly, spleens from mice were collected in 3 mL of RPMI 1640 (Invitrogen, Carlsbad, CA) supplemented with 5% heat-inactivated FBS, then smashed and filtered through 70 µm nylon filters, followed by centrifugation at 500 × g for 5 minutes at room temperature. Red blood cells were then lysed by adding 3 mL of ammonium chloride potassium (ACK) lysis buffer (Invitrogen, Carlsbad, CA, United States) for 4 minutes at room temperature. An equal volume of PBS was added to neutralize the lysis buffer. Cells were centrifuged again at 500 × g for 5 minutes at room temperature and the resulting cell pellets were resuspended in PBS. Splenocytes were then mixed with B16F10 cells in a 1:1 ratio and injected into naïve mice.

### ELISA assay for immune response assessment

The mean optical density (OD) values of total anti-S1 IgG in the serum of immunized mice were determined by enzyme-linked immunosorbent assay (ELISA), as described previously (13). Three weeks post-immunization, serum samples from immunized and control mice were collected for assessment. Briefly, 96-well high-binding plates (Thermo Scientific) were coated with the SARS-CoV-2 S subunit (Sino Biological) at 1 µg/mL in PBS and incubated overnight at 4 °C. Plates were then washed, blocked with a 5% skim milk solution and incubated with a 10-fold serial dilution of mouse sera. After washing, peroxidase-conjugated rabbit anti-mouse IgG secondary antibodies (Jackson Immunoresearch Laboratories) were added and color development was initiated using 3,3′,5,5′-tetramethylbenzidine (TMB) substrate (KPL, Gaithersburg, MD). The reactions were stopped with 0.16 M sulfuric acid and absorbance was measured at 450 nm using a spectrophotometer.

### Statistical analysis

Statistical analyses and graphical presentations in this study were performed using GraphPad Prism version 10 (GraphPad Software, Inc., La Jolla, CA, United States). Multigroup comparisons were conducted using Two-way Analysis of Variance (ANOVA). All values are presented as the mean ± SEM. As tumor volumes may exhibit right-skewed distributions, two-way ANOVA results were interpreted alongside individual tumor growth curves and survival outcomes.

Survival differences between tumor-bearing and treated mice were assessed using Kaplan-Meier survival curves and the Log-rank (Mantel-Cox) test was applied for analysis. Statistical significance is reported as: ∗P ≤ 0.05, ∗∗P ≤ 0.01, ***P ≤ 0.001 and ****P ≤ 0.0001.

## Results

### Intratumoral administration of SARS-CoV-2-based vaccines suppresses tumor growth and prolongs survival in a murine melanoma model

To explore the therapeutic potential of redirecting antiviral immunity toward tumors, we evaluated the antitumor efficacy of SARS-CoV-2 vaccines in a melanoma mouse model. Tumor growth and survival were assessed in C57BL/6J mice bearing B16F10 melanoma tumors following treatment with two distinct platforms: pVAX-SARS-S, a DNA vaccine encoding the SARS-CoV-2 Spike protein S1 subunit (13), or rAd-SARS2-S1/CD40L, a recombinant adenovirus serotype 5 engineered to express a secreted S1–murine CD40L fusion protein.

Prior to therapeutic assessment, the immunogenicity of both vaccine constructs was confirmed in naïve C57BL/6J mice (n=6 per group) (**Supplementary Figure S1**). Three weeks post-vaccination, both vaccine platforms elicited robust SARS-CoV-2-specific humoral responses, as evidenced by S-specific IgG levels measured by ELISA (**Supplementary Figure S1C**).

**Figure 1:**
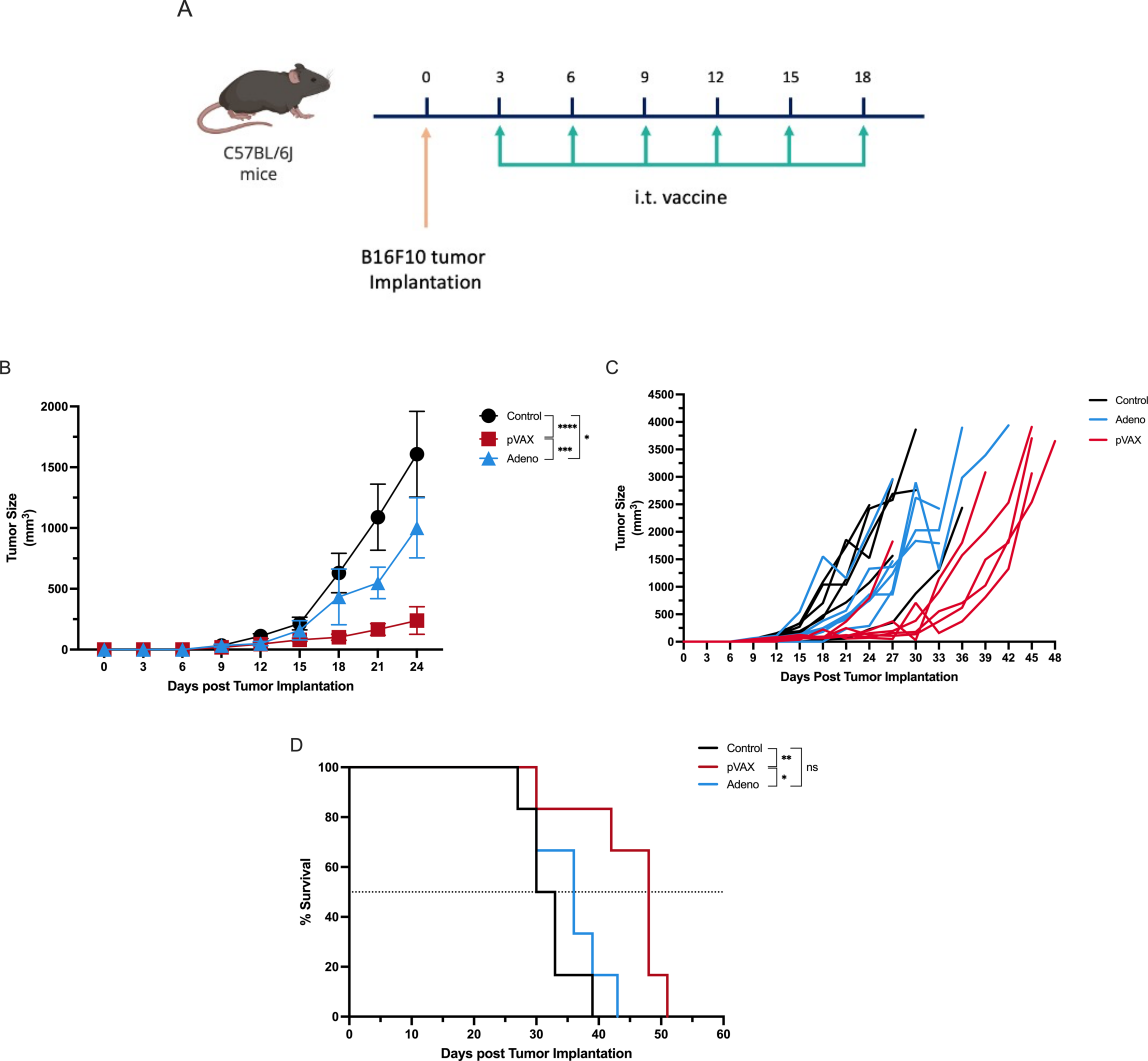
Intratumoral vaccination with SARS-CoV-2-based constructs reduces tumor growth and prolongs survival in melanoma-bearing mice. **(A)** Schematic of the experimental design showing B16F10 tumor implantation on day 0 and intratumoral injections of PBS, pVAX-SARS-S, or rAd-SARS2-S1/CD40L every three days on days 3, 6, 9, 12, 15 and 18. **(B)** Tumor volume measurements over time following treatment. **(C)** Individual tumor growth kinetics in each group. **(D)** Kaplan–Meier survival analysis of treated mice. ns = not significant. *P < 0.05, **P < 0.01, ***P < 0.001, ****P < 0.0001.

To assess therapeutic efficacy, naïve C57BL/6J mice were inoculated intradermally with B16F10 melanoma cells and treated with six intratumoral doses of PBS, pVAX-SARS-S, or rAd-SARS2-S1/CD40L administered between days 3 and 18 (**Figure 1A**). Tumor growth was monitored every three days and survival was recorded until humane endpoints were reached. By day 24, pVAX-treated mice showed a significant inhibition of tumor growth (mean = 238.7 mm³) compared to control mice (1607 mm³; P < 0.0001). Tumors in the rAd group averaged 1001 mm³, which was significantly smaller than control (*P* < 0.05), but significantly larger than in the pVAX group (*P* < 0.001) (**Figure 1B**). Individual tumor growth curves showed consistent responses in the pVAX group and more variation in the rAd group (**Figure 1C**). Survival analysis showed that pVAX-SARS-S significantly improved survival compared to control (median: 48 vs. 31.5 days; P < 0.01). There was no significant difference between rAd-SARS2-S1/CD40L and control (36 vs. 31.5 days; not significant), while pVAX-treated mice survived significantly longer than those treated with rAd (P < 0.05) (**Figure 1D**).

These results indicate that intratumoral administration of pVAX-SARS-S delays tumor progression and extends survival in a melanoma model. The rAd-SARS2-S1/CD40L construct showed modest therapeutic activity under the same conditions.

### Extended intratumoral vaccine therapy reduces tumor growth and enhances survival in a naïve melanoma mouse model

**Figure 2:**
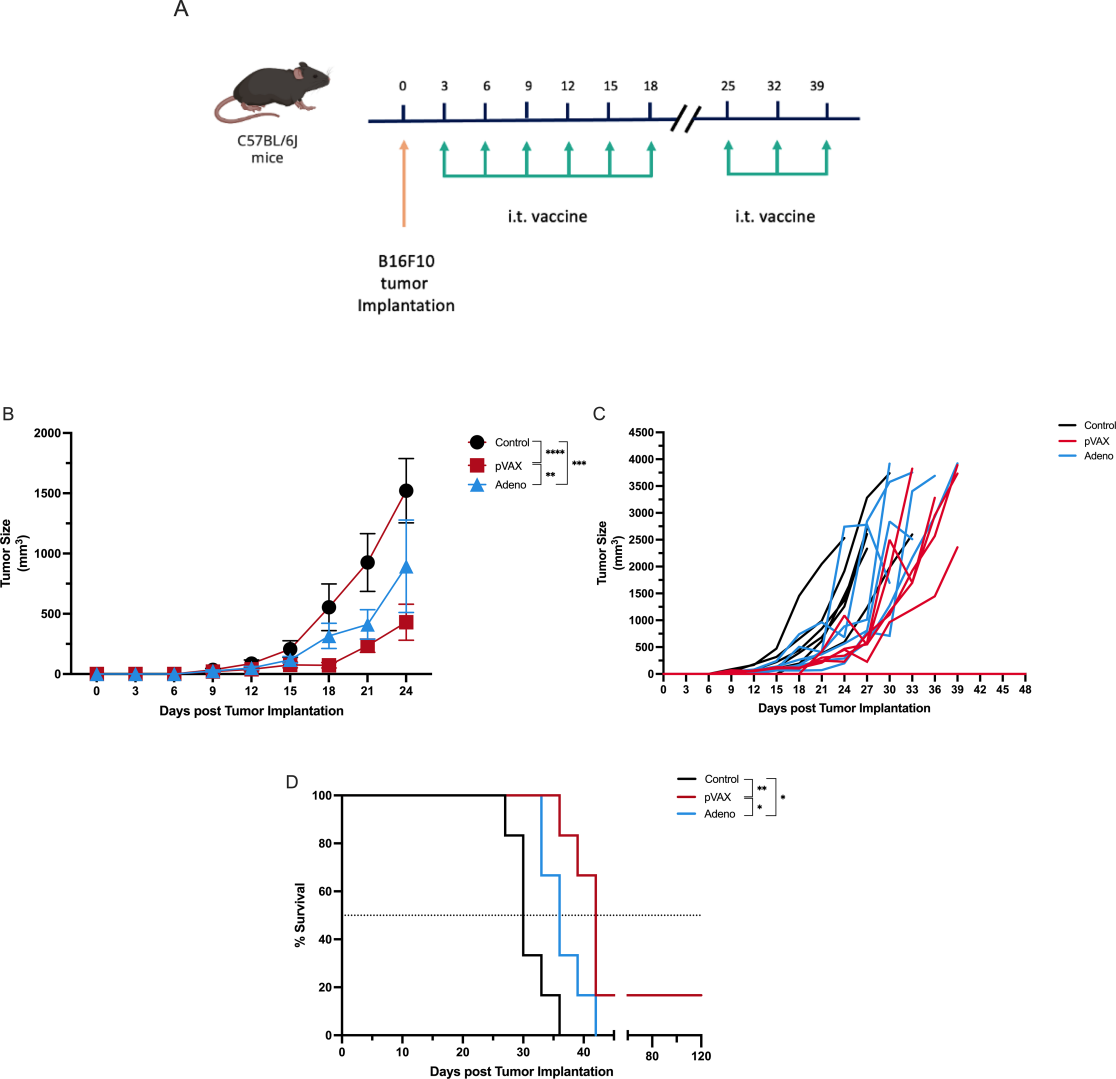
Extended intratumoral vaccine delivery enhances tumor control and survival in melanoma-bearing mice. **(A)** Experimental design showing B16F10 implantation followed by intratumoral injections of PBS, pVAX-SARS-S, or rAd-SARS2-S1/CD40L. The treatment was administered in two cycles: Cycle 1 included injections every three days on days 3, 6, 9, 12, 15 and 18 post-implantation, while Cycle 2 consisted of weekly injections on days 25, 32 and 39. **(B)** Tumor volume measurements over time. **(C)** Individual tumor growth kinetics for each group. **(D)** Kaplan–Meier survival analysis. *P < 0.05, **P < 0.01, ***P < 0.001, ****P < 0.0001.

To evaluate the effect of prolonged intratumoral vaccine delivery, we used the same murine melanoma model and administered two rounds of intratumoral therapy following tumor implantation. C57BL/6J mice (n = 6 per group) were injected intradermally with B16F10 melanoma cells on day 0. Mice then received intratumoral vaccine injections of PBS (control), pVAX-SARS-S, or rAd-SARS2-S1/CD40L on days 3, 6, 9, 12, 15 and 18, followed by a second round of injections on days 25, 32 and 39 (**Figure 2A**). Tumor growth was measured every three days and survival was recorded until endpoint.

By day 24, mice treated with pVAX-SARS-S showed the lowest mean tumor burden (429.7 mm³), followed by the rAd-SARS2-S1/CD40L group (894.2 mm³), while tumors in the control group reached a mean of 1521 mm³. Both vaccine-treated groups exhibited significant tumor growth delay relative to control (*P* < 0.0001 for pVAX; *P* < 0.001 for rAd) and tumor size was also significantly lower in the pVAX group compared to rAd (*P* < 0.01) (**Figure 2B**). Individual tumor growth curves confirmed more uniform and sustained control in the pVAX group and a partial but clear effect in the rAd group (**Figure 2C**).

Survival analysis supported the tumor growth findings. Median survival was 30 days for control mice, 36 days for rAd-treated mice and 42 days for pVAX-treated mice. Survival was significantly improved in both treatment groups compared to control (*P* < 0.01 for pVAX; *P* < 0.05 for rAd) and mice in the pVAX group also lived significantly longer than those in the rAd group (*P* < 0.05) (**Figure 2D**).

These results indicate that both vaccine platforms are therapeutically active when administered repeatedly into tumors. The pVAX-SARS-S construct provided stronger and more durable control, but rAd-SARS2-S1/CD40L also showed significant efficacy under extended dosing conditions.

### Pre-existing SARS-CoV-2 immunity augments the therapeutic efficacy of intratumoral pVAX-SARS-S vaccination in melanoma model

**Figure 3:**
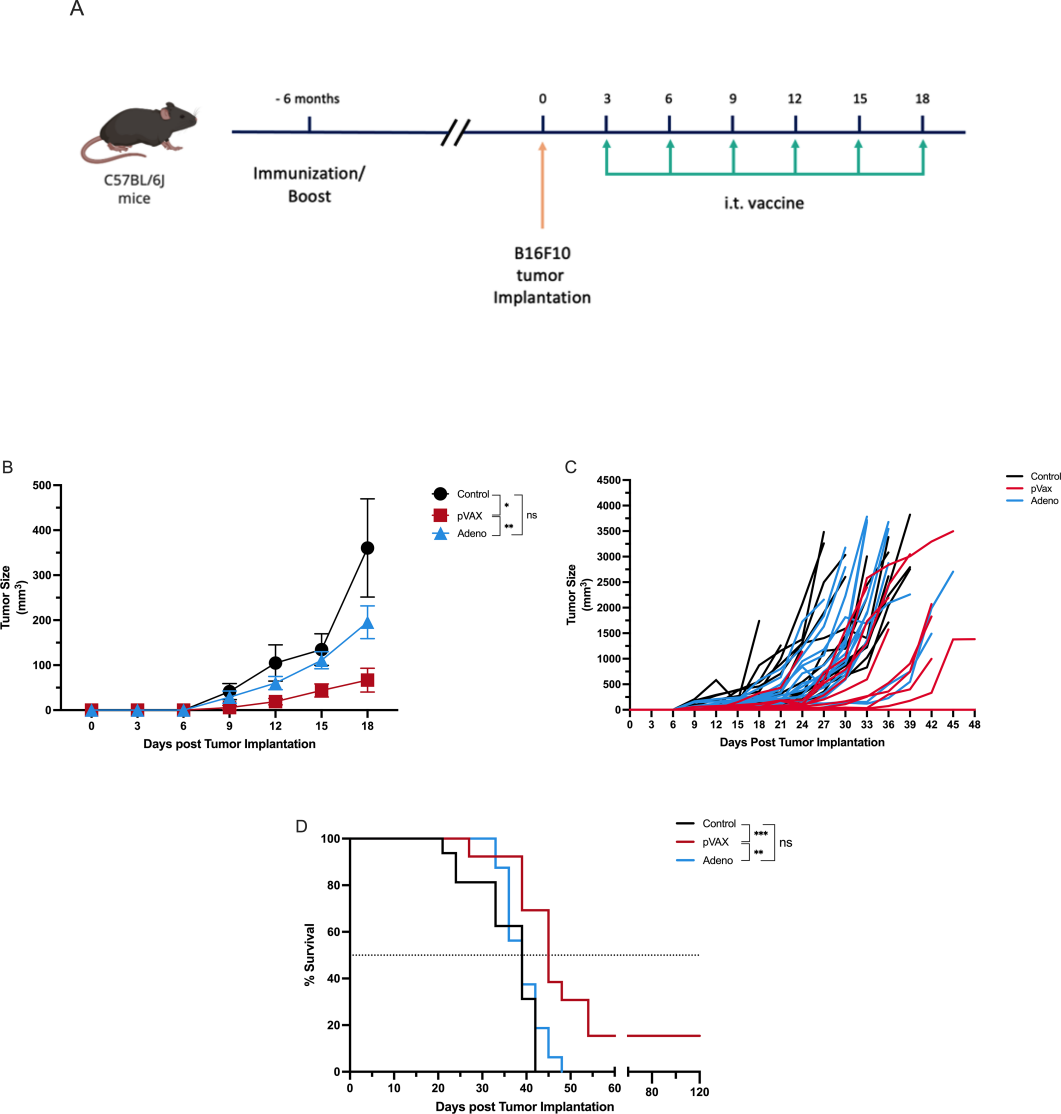
Pre-existing SARS-CoV-2 immunity enhances the antitumor efficacy of intratumoral DNA vaccination. **(A)** Experimental design showing immunization with pVAX-SARS-S, rAd-SARS2-S1/CD40L, or PBS six months prior to B16F10 implantation, followed by intratumoral vaccination every three days on days 3, 6, 9, 12, 15 and 18. **(B)** Tumor volume measured over time. **(C)** Individual tumor growth kinetics. **(D)** Kaplan–Meier survival analysis of pre-immunized mice. ns = not significant. *P < 0.05, **P < 0.01, ***P < 0.001.

To determine whether pre-existing SARS-CoV-2 immunity enhances antitumor efficacy, C57BL/6J mice were immunized with either pVAX-SARS-S (n=12) or rAd-SARS2-S1/CD40L (n=16) six months prior to tumor implantation. Control mice (n=16) received PBS. All groups were then challenged with B16F10 melanoma cells, followed by six intratumoral vaccine doses on days 3, 6, 9, 12, 15 and 18 post-implantation (**Figure 3A**).

Tumor growth was assessed every three days. On day 18, pVAX-immunized mice showed significantly reduced tumor volume compared to controls (66.7 mm³ vs. 360.5 mm³; *P* < 0.05) and tumor volume in the pVAX group was also significantly smaller than in the rAd group (*P* < 0.01). Tumor size in the rAd group was not significantly different from the control group (**Figure 3B**). Individual tumor growth curves showed consistent tumor control in the pVAX group, with more variable responses in the rAd and control groups (**Figure 3C**).

Survival analysis demonstrated a significant increase in survival in the pVAX group compared to control (*P* < 0.001). Survival in the rAd group was not significantly different from the control group. However, pVAX-treated mice survived significantly longer than rAd-treated mice (P < 0.01) (**Figure 3D**).

These findings indicate that pre-existing immunity elicited by the pVAX-SARS-S vaccine enhances the antitumor effect of subsequent intratumoral vaccination. In contrast, pre-immunization with rAd-SARS2-S1/CD40L did not provide a measurable therapeutic advantage under the same conditions.

#### Extended intratumoral vaccine administration improves tumor control and survival in pre-immunized mice with established melanoma

**Figure 4:**
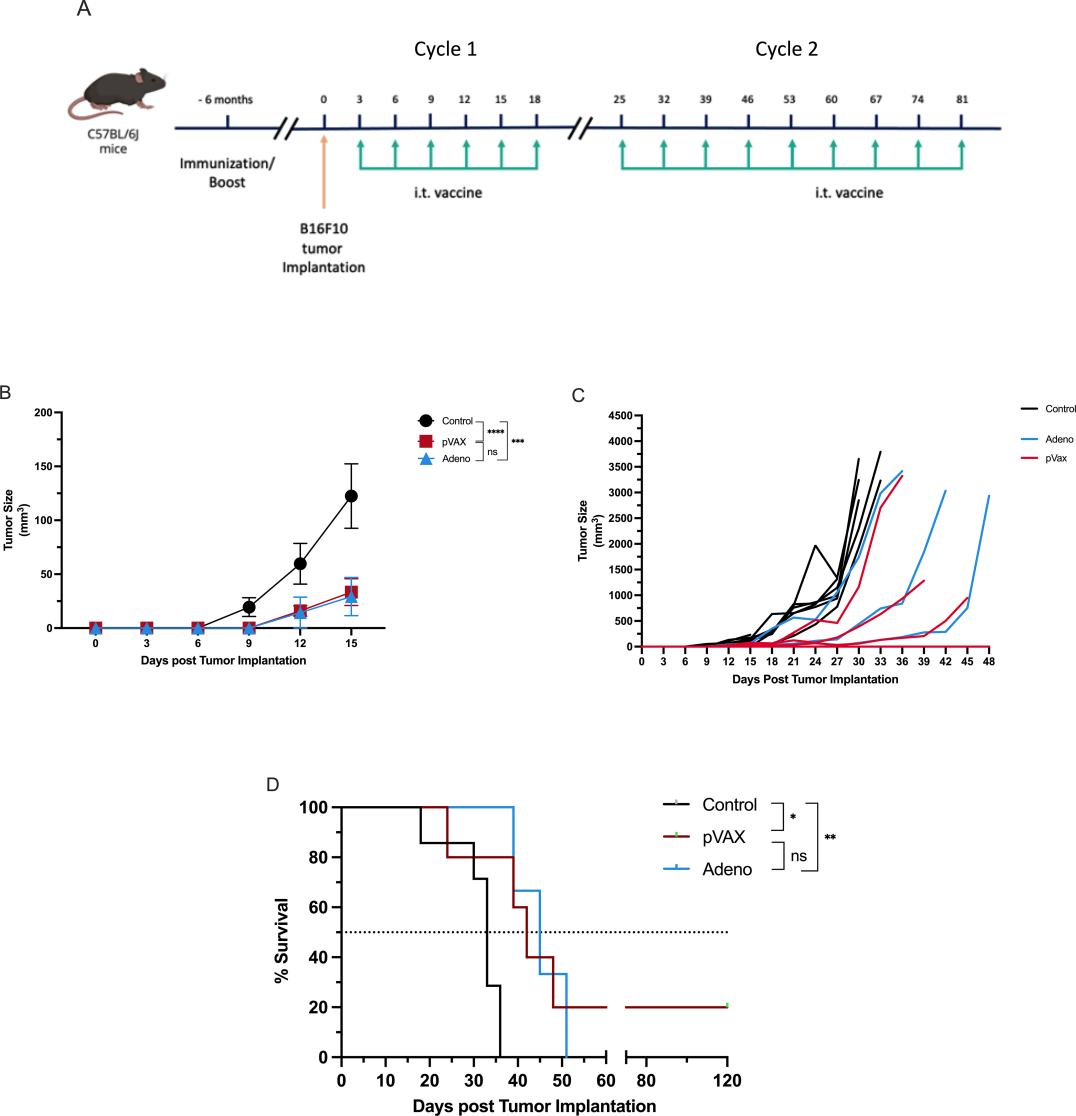
Extended intratumoral vaccination improves therapeutic efficacy in pre-immunized melanoma-bearing mice. **(A)** Schematic of the two-cycle dosing strategy in mice immunized six months prior. Cycle 1 consisted of injections administered every three days on days 3, 6, 9, 12, 15 and 18 post-tumor implantation, while Cycle 2 involved weekly injections on days 25, 32, 39, 53, 67 and 81. **(B)** Tumor volume measured over time. **(C)** Individual tumor growth kinetics. **(D)** Kaplan–Meier survival analysis. ns = not significant. *P < 0.05, **P < 0.01, ***P < 0.001, ****P < 0.0001.

To evaluate whether extending intratumoral vaccine administration enhances therapeutic benefit in the context of pre-existing SARS-CoV-2 immunity and to assess the durability of responses observed in earlier experiments, we used a two-cycle dosing strategy in mice that had been pre-immunized six months earlier with either pVAX-SARS-S (n=5) or rAd-SARS2-S1/CD40L (n=3). Control mice (n=7) received PBS. All mice were implanted intradermally with B16F10 melanoma cells and treated intratumorally with their respective vaccines (or PBS) on days 3, 6, 9, 12, 15 and 18 (cycle 1), followed by additional booster injections on days 25, 32, 39, 53, 67 and 81 (cycle 2) (**Figure 4A**).

By day 15, tumor volumes were significantly lower in both treatment groups compared to controls. Mice receiving pVAX-SARS-S had a mean tumor volume of 33.37 mm³, while those treated with rAd-SARS2-S1/CD40L had a mean of 29.31 mm³. In contrast, control mice exhibited significantly larger tumors (mean = 122.4 mm³). The differences were statistically significant for both treatment groups compared to control (*P* < 0.0001 for pVAX; *P* < 0.001 for rAd), but not between the two vaccine groups (**Figure 4B**). Individual tumor trajectories showed consistent suppression in pVAX-treated mice and more variable control in the rAd group (**Figure 4C**). Extended treatment also led to improved survival. Median survival was 47 days for the pVAX group and 45 days for the rAd group, compared to 33 days in the control group. Survival was significantly prolonged in both vaccinated groups relative to control (*P* < 0.05 for pVAX; *P* < 0.01 for rAd), with no significant difference observed between pVAX and rAd (**Figure 4D**).

These findings indicate that extended intratumoral dosing enhances the antitumor efficacy of both SARS-CoV-2 vaccine constructs in mice with prior immunization. The pVAX-SARS-S DNA vaccine maintained consistent tumor control and survival benefit, while rAd-SARS2-S1/CD40L also demonstrated therapeutic activity under the extended schedule.

#### Local intratumoral vaccination confers systemic protection in pre-immunized melanoma mice

**Figure 5:**
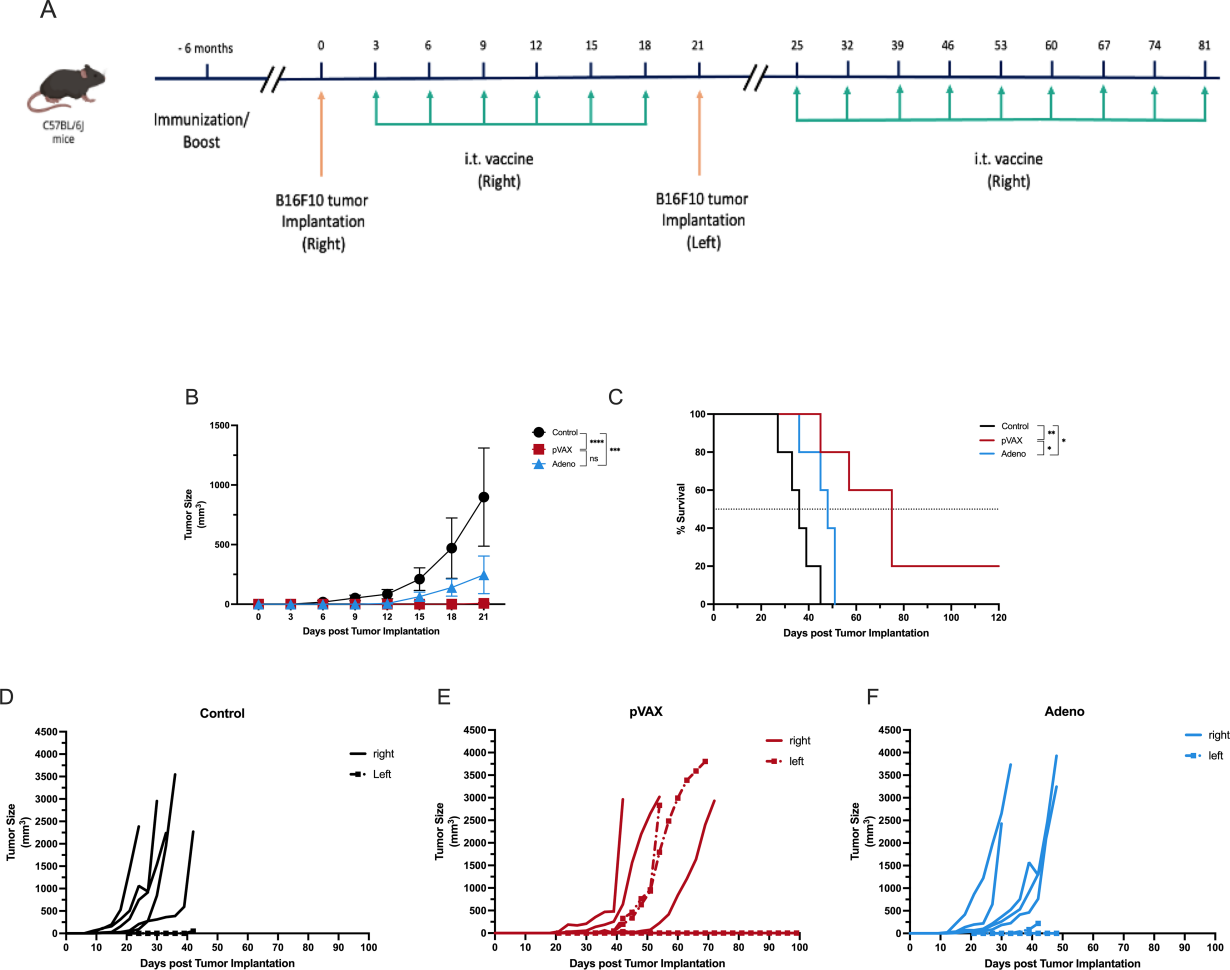
Local intratumoral vaccination confers systemic protection in a bilateral flank tumor model. **(A)** Experimental design showing initial B16F10 tumor challenge and intratumoral vaccination in the right flank, followed by secondary tumor implantation in the left flank on day 21. **(B)** Tumor volume measured over time in the injected (right) flank. **(C)** Kaplan–Meier survival analysis. **(D–F)** Individual tumor growth kinetics in both flanks for each group: **(D)** PBS, **(E)** pVAX-SARS-S and **(F)** rAd-SARS2-S1/CD40L. ns = not significant. *P < 0.05, **P < 0.01, ***P < 0.001, ****P < 0.0001.

To determine whether extended intratumoral vaccine administration could induce systemic antitumor immunity, we conducted a bilateral flank tumor model in pre-immunized mice. C57BL/6J mice (5 mice/group) were vaccinated six months prior with pVAX-SARS-S, rAd-SARS2-S1/CD40L, or PBS. All mice were first challenged with B16F10 melanoma cells in the right flank and received intratumoral vaccine injections at that site for 18 days (**Figure 5A**). On day 21, mice received a second B16F10 tumor inoculation in the contralateral (left) flank, while intratumoral vaccination continued in the original (right) tumor only.

On day 21, tumor volume was measured in the injected (right) flank. Mice treated with pVAX-SARS-S exhibited significantly reduced tumor growth compared to controls (mean volume: 7.3 mm³ vs. 898.6 mm³; *P* < 0.0001), while rAd-SARS2-S1/CD40L also significantly suppressed tumor volume relative to PBS controls (245.9 mm³; *P* < 0.001). No significant difference was observed between the pVAX and rAd-treated groups (**Figure 5B**).

Survival analysis showed that pVAX-treated mice had a significantly prolonged survival compared to both control (*P* < 0.01) and rAd-treated groups (*P* < 0.05). The survival difference between rAd and control groups was not statistically significant (**Figure 5C**). Individual tumor growth curves in both flanks for each group further support these findings. Mice in the control group (**Figure 5D**) showed rapid tumor progression in both flanks. In contrast, pVAX-treated mice (**Figure 5E**) exhibited controlled tumor growth in both the injected and non-injected sites. The rAd group (**Figure 5F**) showed partial control in the left flank compared to controls, though less pronounced than in the pVAX group.

These data suggest that sustained intratumoral vaccine administration in previously immunized mice can elicit a systemic antitumor response, capable of controlling tumors at distant, untreated sites.

#### Adoptive transfer of splenocytes from vaccinated mice suppresses tumor growth and improves survival in naïve recipients

**Figure 6:**
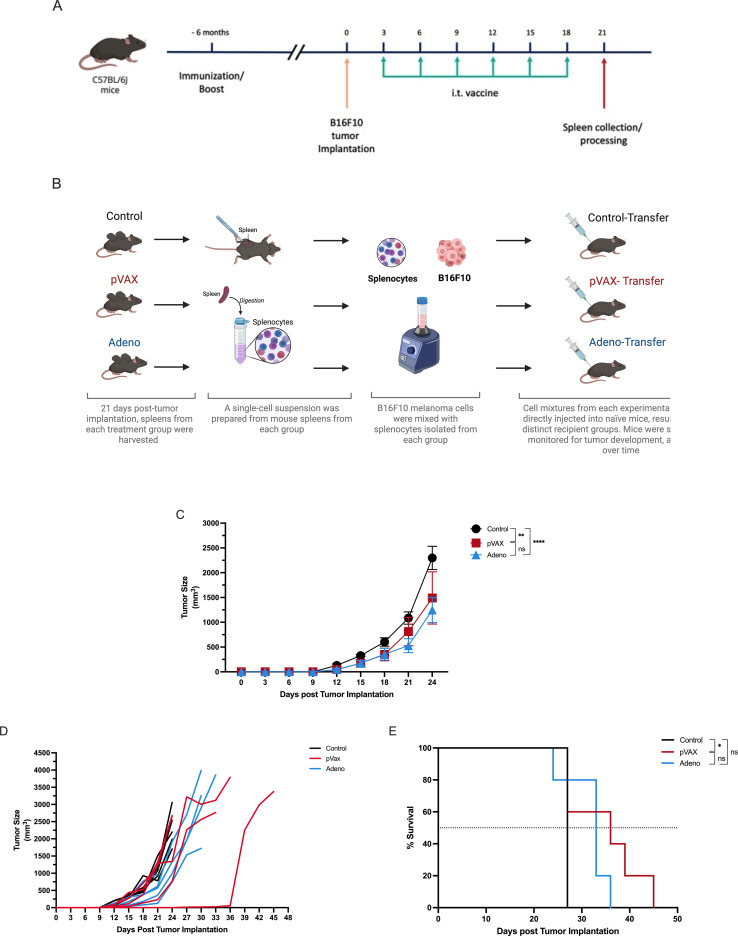
Adoptive transfer of splenocytes from vaccinated, tumor-bearing mice delays tumor growth and improves survival in naïve recipients. **(A)** Schematic of the adoptive transfer design. Donor mice were immunized 6 months prior to B16F10 tumor implementation and then treated intratumorally every three days on days 3, 6, 9, 12, 15 and 18. Spleens were collected from individual donors on day 21. **(B)** Collected splenocytes were mixed 1:1 with B16F10 tumor cells before injection into naïve recipients. **(C)** Tumor volume measured in recipient mice over time. **(D)** Individual tumor growth kinetics. **(E)** Kaplan–Meier survival analysis of recipient mice. ns = not significant. *P < 0.05, **P < 0.01, ****P < 0.0001.

To evaluate whether splenocytes from tumor-bearing vaccinated mice could transfer protective antitumor immunity, we performed an adoptive transfer experiment. First, C57BL/6J donor mice (n=5/group) were immunized with pVAX-SARS-S, rAd-SARS2-S1/CD40L, or PBS. All donor mice were then implanted with B16F10 melanoma cells and received six intratumoral vaccine or PBS injections on days 3, 6, 9, 12, 15 and 18 (**Figure 6A**).

On day 21, spleens were harvested from individual donor mice. As shown in **Figure 6B**, splenocytes from each donor were processed separately, mixed at a 1:1 ratio with B16F10 tumor cells and injected into a naïve recipient mouse (n=5/group). This strategy allowed us to directly test whether immune cells, particularly activated and memory T cells, generated by intratumoral vaccination could control tumor growth without further intervention. By mixing splenocytes with tumor cells prior to implantation, we created a setting in which any delay in tumor outgrowth would reflect the functional capacity of the transferred immune cells.

By day 24, tumor volumes in recipient mice were significantly reduced in both pVAX and rAd groups compared to PBS controls (~1,400 mm³ for pVAX, ~1,200 mm³ for rAd, vs. ~2,300 mm³ for control). These reductions were statistically significant (*P* < 0.01 for pVAX; *P* < 0.0001 for rAd), although there was no significant difference between the two vaccine groups (**Figure 6C**).

Survival analysis showed that splenocytes from pVAX-treated donors conferred a modest but significant survival advantage (*P* < 0.05). On the other hand, splenocytes from rAd-treated mice and control mice did not significantly affect survival (**Figure 6E**).

These results demonstrate that splenocytes from vaccinated, tumor-bearing mice can partially control tumor growth upon transfer to naïve hosts. The ability of pVAX-derived splenocytes to extend survival suggests that this vaccine generates a more durable and functionally active antitumor immune response compared to rAd under these conditions.

## Discussion

Neoadjuvant immunotherapy offers a viable method for maximizing the benefits of immunotherapy while avoiding off-target effects. Direct immune-stimulating injections into the tumor prime the local immune system to produce a systemic, long-lasting clinical response. Several attempts have been made to use viruses to target cytopathic effects against cancer. Replication-defective viral vectors and replication-competent oncolytic viruses may both function primarily by inducing more potent antitumor immune responses, according to a growing body of research. The virus’s potency can be increased when its genome contains genes that stimulate an immune response, such as cytokines and costimulatory proteins. In our study, we have revealed that intratumoral administration of SARS-CoV-2 vaccines induces local immune activation within the TME that contributes to antitumor responses.

In this study, we have focused on using different SARS-CoV-2 vaccines to provide viral immunity that can be utilized in stimulating anti-cancer immune responses. The rAd-SARS-S/CD40L vaccine is an adenovirus vector vaccine that encodes the SARS-CoV-2 spike glycoprotein (S1) fused to CD40 ligand (CD40L) and the plasmid DNA vaccine encodes the SARS-CoV-2 spike glycoprotein (S). Both vaccines successfully stimulated a humoral immune response. Intratumoral vaccine injection with pVAX-SARS-S vaccine generated better treatment outcomes regarding tumor volume and survival rate in the melanoma mouse model compared to control and rAd-SARS-S/CD40L vaccinated mice. The effect of pVAX-SARS-S intratumoral vaccine injection was significant both in the presence and absence of pre-existing SARS-CoV-2 immunity. The pVAX-SARS-S has been shown to induce long-term Th1-skewed cellular immunity and humoral immunity in mice (13). This was indicated by the significant levels of binding IgG antibodies, neutralizing antibodies (nAbs) and cytokine (IFN-γ, TNF and IL-2) production from both memory CD4^+^ and CD8^+^ T cells. On the other hand, rAd-SARS-S/CD40L vaccine showed enhanced survival rate and reduced tumor volume only with prolonged intratumoral vaccine injection. This study is intended as a proof-of-concept demonstrating that intratumoral vaccination can locally harness pre-existing antiviral immunity to promote antitumor responses, without implying direct antigen-specific redirection of SARS-CoV-2 immunity toward tumor antigens.

Furthermore, adoptive transfer of splenocytes from mice immunized with pVAX-SARS-S resulted in a marked anti-tumor effect and significantly prolonged survival. However, the transfer of splenocytes from the rAd-SARS2-S1/CD40L vaccinated mice significantly reduced tumor growth in naïve mice, but did not improve the survival rate significantly. The immune system’s response to both the tumor and the treatment can play a significant role in determining survival outcomes. An inadequate or suppressed immune response may lead to poor survival outcomes despite reductions in tumor volume. Additionally, the treatment might reduce the size of the tumor but leave behind dormant cancer cells or a small population of resistant cells that can later proliferate and cause relapse. Thus, exploring the TME and vaccine-induced immune response will be important to better understand and optimize the therapeutic outcomes of intratumoral SARS-CoV-2 vaccine therapy.

Remarkably, splenocyte transfer from immunized mice was sufficient to generate an antitumor response in naïve mice, indicating that immune cells from the treated mice can, in fact, provide an antitumor response in naïve mice. The TME in most melanoma tumors contains tumor-infiltrating lymphocytes (TILs) that express PD-L1, mediating immune suppression. Further characterization of lymphocyte phenotypes and cytokine profiles generated from the SARS-CoV-2 immunized mice splenocytes will provide insight into the mechanism of antitumor response generated in the naïve mice (14). The ultimate goal of intratumoral immunotherapy is to provoke immunologically cold tumors and trigger immune cells at the tumor site that subsequently provide a systemic antitumor immune response. We have provided evidence that pre-existing SARS-CoV-2 immunity through vaccination can contribute to local and systemic antitumor responses.

Moreover, this study indicates that intratumoral vaccine injection with SARS-CoV-2 vaccines can generate an antitumor response with or without pre-existing SARS-CoV-2 immunity for pVAX-SARS-S. Nevertheless, pre-existing SARS-CoV-2 immunity can be locally leveraged to promote tumor inhibition and enhance survival rates in the melanoma mouse model. Therefore, intratumoral vaccine injections of a non-oncolytic viral vaccine, such as a SARS-CoV-2-based vaccine platform, can serve as a local immune stimulus that contributes to anti-cancer immune responses.

## Conclusion

Our findings suggest that intratumoral vaccine injections of SARS-CoV-2 vaccines can reduce tumor growth and enhance survival in the melanoma mouse model. Additionally, the generated immune response can provide local and systemic antitumor effects, which were sufficient to influence distal tumor growth and could be transferred to naïve mice, generating an antitumor response against induced melanoma tumors. For intratumoral immunotherapy, the most desirable approach would be a tumor-agnostic treatment strategy that is effective in providing both local and systemic antitumor responses, compatible with large-scale production. *In-situ* vaccination, in which intratumoral administration of immune-stimulating agents provokes local immune activation within the TME, is well aligned with these criteria.

One of the major challenges remains the translation of the preclinical findings into the clinical setting (15, 16). Thus, intratumoral vaccine injection is still in the early stages of development, but it has the potential to be a promising effective cancer immunotherapy approach. Further research is needed to determine the safety and effectiveness of the vaccine and to explore the underlying local and systemic immune mechanisms in future translational and clinical studies.

## Data Availability

The original contributions presented in the study are included in the article/**Supplementary Material**. Further inquiries can be directed to the corresponding authors.

## Supplementary material

Supplementary Figure 1 Humoral immune response in vaccinated mice. (A-B) Schematic of the immunization strategy showing administration of rAd-SARS2-S1/CD40L (A) or pVAX-SARS-S (B) in mice, followed by serum collection three weeks post the final booster dose (on day 63). (C) ELISA measurement of SARS-CoV-2 S1-specific IgG antibody titers in the serum of vaccinated mice. Data are presented as mean ± SEM. *P < 0.05, **P < 0.01, ***P < 0.001, ****P < 0.0001.
