# Supplementary figures and images for: Harnessing SARS-CoV-2 immunity to promote antitumor responses through intratumoral vaccination and adoptive transfer
Source: Front Immunol. 2026 Feb 23;17:1711569. doi: 10.3389/fimmu.2026.1711569 (PMC12968191; doi:10.3389/fimmu.2026.1711569)

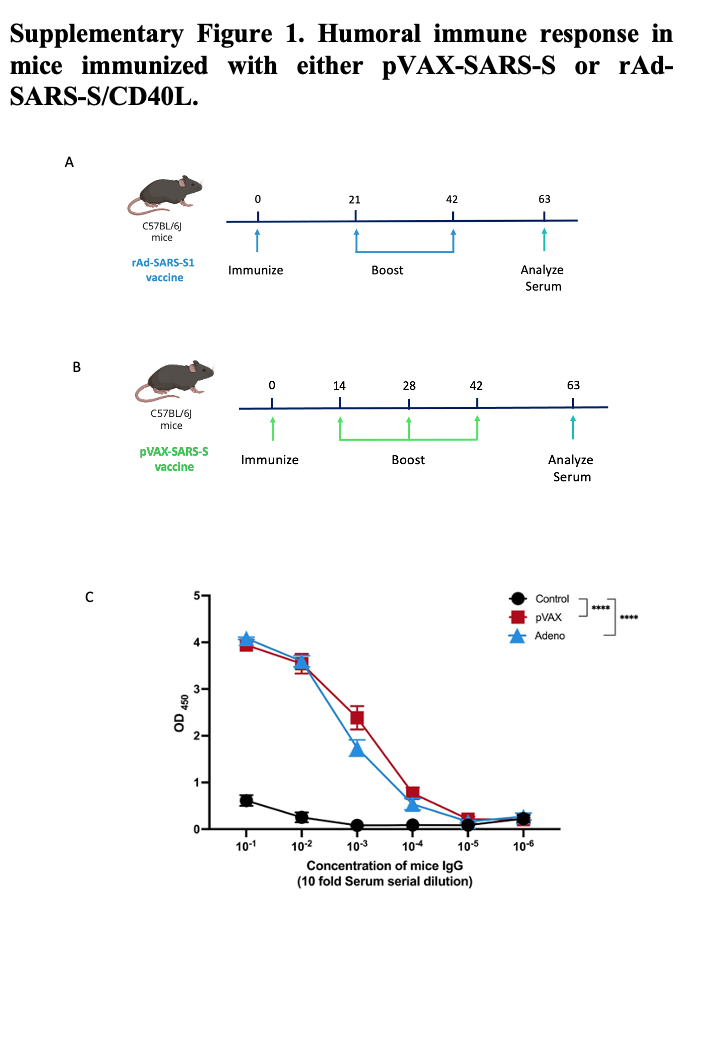

Supplement: Supplementary Figure 1 — Humoral immune response in vaccinated mice. (A-B) Schematic of the immunization strategy showing administration of rAd-SARS2-S1/CD40L (A) or pVAX-SARS-S (B) in mice, followed by serum collection three weeks post the final booster dose (on day 63). (C) ELISA measurement of SARS-CoV-2 S1-specific IgG antibody titers in the serum of vaccinated mice. Data are presented as mean ± SEM. *P < 0.05, **P < 0.01, ***P < 0.001, ****P < 0.0001. [file Image1.tiff]
